# Pediatric Oncology Hospice: A Comprehensive Review

**DOI:** 10.1177/10499091241227609

**Published:** 2024-01-15

**Authors:** Ali Tafazoli, Katharine Cronin-Wood

**Affiliations:** 1 Healthcare administration program, 25480 St Lawrence College, Kingston Campus, ON, Canada; 2 Hospice Kingston, 4257 Queen’s University, Kingston, ON, Canada; 3 Department of Biomedical and Molecular Sciences, 4257 Queen’s University, Kingston, ON, Canada

**Keywords:** cancer, child, hospice, neoplasms, palliative care, review

## Abstract

Pediatric hospice is a new terminology in current medical literature. Implementation of pediatric hospice care in oncology setting is a vast but subspecialized field of research and practice. However, it is accompanied by substantial uncertainties, shortages and unexplored sections. The lack of globally established definitions, principles, and guidelines in this field has adversely impacted the quality of end-of-life experiences for children with hospice needs worldwide. To address this gap, we conducted a comprehensive review of scientific literature, extracting and compiling the available but sparse data on pediatric oncology hospice from the PubMed database. Our systematic approach led to development of a well-organized structure introducing the foundational elements, highlighting complications, and uncovering hidden gaps in this critical area. This structured framework comprises nine major categories including general ideology, population specifications, role of parents and family, psychosocial issues, financial complications, service locations, involved specialties, regulations, and quality improvement. This platform can serve as a valuable resource in establishing a scientifically reliable foundation for future experiments and practices in pediatric oncology hospice.

## Introduction

The institution of modern hospice care (HC) dates back to 1967, when the first hospice clinic opened in London, England and the first US hospice home-based service was founded in 1974.^1^ These outstanding institutions were designed exclusively to serve adults with terminal and debilitating conditions with certain illnesses. This unique field of medicine has grown gradually, embracing a broader spectrum of patients, numerous specialized organizations, hospital wards, voluntary groups and allied health professionals. However, it took many years for the establishment of child-centered services, even in developed countries.^2^ The first North American free-standing pediatric hospice center was established in 1995, in British Columbia, Canada.^3^ This fact shows the lagging process of knowledge and practice transference from adult setting to pediatrics, considering the importance of pediatric hospice care (PHC) as an exclusive medical entity.

Neoplastic disorders are among the leading causes of death in pediatric populations.^4,5^ Accordingly, a majority of applicants for pediatric hospice includes those families affected by childhood cancers. There are some features in the group of children with cancer that makes them contrast with populations suffering from other life-threatening diseases, at least to some extent. Patients in this category commonly and chronically experience pain, somatic symptoms, disturbed mood, excessive fear and anxiety, lack of resilient, self-worth issues besides severe coping challenges due to their age-related limitations. Also, with a sick child who is dying of cancer all the other members of the family may suffer in a chronic way, resulting in a huge load of stress in their mutual life time or afterwards.^6^ In addition, cancer is a unique entity among the human pathologies, regarding the care approaches and outcomes. There is a divergent spectrum of malignancies with different, and sometimes unpredictable cure rates in pediatrics. The advent of novel techniques has changed the expectations of survival in the affected patients.^7^ This fact, increased the level of challenges and uncertainties in this field.

Considering the complications and hurdles in pediatric hospice and childhood oncology the requirement of pediatric-plus-oncology care as a co-specialty in this common ground, makes establishing the facilities for these groups more challenging, compared to the care level provided to each setting, separately. Pediatric oncology hospice is generally a new terminology in current medical literature.^6^ Despite the fact that it involves a very wide spectrum of services, specialties and target populations, the infrastructures for providing adequate and efficient care are still debatable. Even the terminology to address the issue is not well-defined in medical literature.^8^ Besides several other aetiologies, these factors cause many end stage children with cancer to undergo frequent futile aggressive therapies, all over the world. Hence, they experience a considerable level of suffering in their last days, without adequate symptom control. ^4^ Unfortunately, many countries around the world are deprived from such significantly vital frameworks. In fact, different geographic territories have a very diverse range for quality and quantity of hospice palliative care, globally.^9^ This defect stems from different underlying causes including economic, academic and cultural shortcomings. Also, governments have a big role in adopting these specialized care strategies in the national health plans.^10^ This observation is more true about certain locations in Africa,^11^ Asia^12,13^ and South America.^14^ Lack of knowledge and experience in the form of education and goal-directed studies, can be a prominent etiology in this worldwide inadequacy. Otherwise, children have always been cherished and supported from almost all the officially accredited cultural, national, and global parties. For example, a systematic analysis reported a global funding for childhood cancer research around US$2 billion in the United States in less than a decade.^15^ Also, in China, provincial governments cover 50 to100% of medical expenses for pediatric cancer patients.^16^

It can be stated that the dimensions of a standard PHC have not been precisely defined, yet.^17-19^ Complexity of the situation in pediatric oncology boosts the level of uncertainties for providing hospice care. In this review, we tried to gather, categorize, and utilize the available, but sparse and limited data about pediatric hospice with a focus on cancer-related hospice care. However, the results and discussions are widely applicable for PHC in other disabling conditions. Generally, any chronic terminal illness with similar features to cancer including preserved consciousness and pain perception, limited mobility, requirement for invasive procedures, age groups, bodily deformities, mental stress, having a defined illness trajectory and care expenses can be approached in the same way.^20,21^ Serious infections, end stage heart and respiratory diseases, metabolic complications and genetic malformations are among these illnesses. However, the data for several of these entries are not as abundant as for cancer hospice.

Our goals in this research are demonstrating the standard approaches, presenting the key parameters, emphasizing on the gaps and finally, developing a scientifically reliable groundwork for the future experiments and practice in pediatric oncology hospice.

## Methods

In order to achieve a well-founded data pool for our study, we explored the accredited scientific database, PubMed, with this search strategy: ((“Child”[Mesh]) AND (“Hospice Care”[Mesh])) AND “Neoplasms”[Mesh]. The word neoplasm was chosen as a standard MeSH term to embrace all the cancer-oriented terminologies in the database. Choosing child as the MeSH term, reliably gave us articles with pediatric-oriented topics and keywords, as well. Then the yielded journal articles were screened one by one, and our desired information was extracted from the most relevant entries. The categories provided in the following parts are derived from the context of the titles first, and then revising them after exploring the abstracts and full-texts. The articles without accessible full-text or those with foreign language text were backed up by extracted articles from Google Scholar within the same topics. The interchangeability of hospice care and palliative care in some of the studies has also been meticulously considered.

## Results

Searching PubMed with the keywords “child,” “hospice care” and “neoplasms” provided 94 journal articles. Majority of those articles were included in this study and several additional ones were utilized for enhancement of the explanations and support of justifications. Countries of origin for the yielded articles from PubMed searching, based on the affiliations of corresponding authors are United States of America, United Kingdom, Canada, Australia, Germany, Italy, China, Republic of Korea, Japan, Taiwan, India, Greece, Turkey, France, Hong Kong, Denmark, Irland, Finland and Uganda, that confirms the international perspective of this review.

## Discussion

Numerous studies have tried to explore different aspects of pediatric end-of-life (EOL) care in cancer settings.^22,23^ According to the results from our database searching, the first noticeable point is the interchangeable usage of hospice and palliation in many articles. As stated by some institutions like Canadian Hospice Palliative Care Association, “both terms are used to refer to the same thing; however, people often use the term hospice care to describe care that is offered in the community rather than in hospitals.” Some references introduced hospice as a part of palliative care.^23^ Nevertheless, interchangeable use of these two terms with inattention cannot be endorsed. In other words, these two terms cannot be used interchangeably unless we are confident that our conclusions, statements or recommendations will have the same effect on both settings or the settings are combined (hospice/palliative care). The reason for this prohibition underlies in differences between the two settings. Although the definitions in different references are not consistent, the start or trigger points of the care, treatment types, place of care, prognosis and the insurance coverage are dissimilar for hospice and palliative care, despite their considerable overlaps.^23^ For instance, nowadays, hospice services are being provided in many hospitals, too, so it should not be considered as a merely home-based care. Based on definitions provided by American National Institute of Aging the most important differentiating factor is that in many regulatory jurisdictions curative approaches are not being implemented in hospice, while they are totally acceptable in palliative or hospice/palliative care. However, life-sustaining treatments are commonly utilized at hospice level of care.^24^ Therefore, local and situational definitions and concepts should be considered when discussing about this issue.

Hospice services are now available for different groups of patients including those with cancers, AIDS, neurologic disorders, heart or lung diseases and severe rheumatologic complications. While approaches for pediatric oncology cases can be applicable for many other situations, specialized strategic planning might be needed for others like those with multiple complex chronic conditions.^25^ The amount of hospice experience for cancer, is generally more than other ailments. Unfortunately, many centers rarely reported their admissions for pediatric patients, over long periods of time^26^ that has resulted in a major loss of valuable data in this setting.

Screening of the titles, abstracts and full-texts in the list of our references guided us to categorize the whole material into 9 specified categories. These parts are discussed separately under the following subheadings including general approaches, population specifications, role of parents and family, psychosocial issues, financial complications, locations of service, involved specialties, regulations, and quality improvement. The practical applicability and specifications of each hospice feature in pediatric oncology is summarized in Table 1.

**Table 1.** Key Features of the Main Compartments of Pediatric Oncology Hospice.

Key Feature	Specifications	Practical Implication	Key References
General hospice considerations	• Hospice is a holistic philosophy • It is different from palliative care • There are lots of uncertainties in this field • Combination of pediatrics, oncology and hospice care establishes an exclusive field of needs and opportunities	• Hospice is a necessity in all health care settings that provide services to children with cancer • More research and reports are mandatory for development of this discipline in medicine	^27,31,33,34,36^
Demography	Main influential demographic characteristics in this field include age, race and ethnicity, family socioeconomic status, geographic location of residence, cancer type and severity, and accessibility of services	• Recording the demographic information of service users is of great importance • Decision making about the level, components and style of care delivery should include considerations of these parameters	^34,43-45,57^
Parents and family members	• Ethical and legal role of parents or other caregivers is a challenging issue • Guardians commonly suffer from mental, financial and social stress in this setting • Support for family members is as important as for the sick child • Cancer progression and outcome may be poorly comprehended by the family	• Hospice care should involve the whole family, both in decision makings and the need for support • National health strategies should include socio-economic and mental support for the family members • Special attention should be focused on youngster siblings in the trajectory • The support should continue after the death	^81,82,84-86^
Psychosocial issues	• Psychosocial issues can affect both the child and the family • Communications between the patient, family and the healthcare provider are important • Different kinds of psychiatric maladies can affect the bereaved family • Addressing the child’s mental concerns is a challenging issue in end-of-life care	• Inclusion of psychosocial support is an obligation in effective hospice • Oncologists and pediatricians should be trained for such situations • Honesty and clarity should be emphasized in communications of pediatric hospice process • Presence of psychiatrists and psychologists in hospice teams is beneficial • Healthcare team should respect emotional responses of the hospice recipients in decision-making	^76,87-89,91^
Budgeting and insurance	• Hospice is a recourse consuming service • Financial burdens of hospice can be devastating for the families • There is a great disparity for availability of public financial support and insurance policies among the hospice institutions all over the world • The case characteristics are influential in determination of expenses • Different parties are collaborating to provide the financial backup for such services	• Governments should be directly and effectively involved in hospice coverage • Insurance providers should be regulated and oriented regarding the hospice cases • Early communication and consultation with the affected families for financial discussions are mandatory • Charities can be involved in the process to improve the quality, extent and accessibility of hospice services	^70,73,82,96,97^
The place for death	• There is debate regarding the optimal place for the last days • Oncology and intensive care units are commonly occupied by these patients • Many studies endorse home-based care at final stages • Many factors affect the choice for time and place of optimal hospice care • Communication gaps hinders the success of choosing the most rational option	• Homecare for hospice should be prioritized in hospice, while considering the feasibility and family ideologies • Effective education from the healthcare team to the family can improve the selection process for the patient • For each cancer-related medical intervention, eligibility, efficiency and autonomy issues should be considered	^52,62,64,75,104^
Specialists	• A diverse range of specialties are involved in hospice care including nurses, physicians, pediatricians, oncologists, psychologists, social workers, pharmacists, legal authorities and other professionals • Pain management is an important part in cancer hospice that needs knowledgeable specialists	• Hospice care should be based on a multidisciplinary team of specialists • Constant care can be optimally delivered by skilled nurses • Presence of specialist in the fields of pediatrics, oncology and hospice care is necessary • Legal sensitivities of this field require involvement of non-health-related specialists • Healthcare practitioners in the field of pediatric oncology should be trained and oriented regarding the process of on-time referrals to hospice services	^58,59,106,107,109^
Regulations	• Hospice regulations are diversly variable among centers • The regulations are evolving • Both cancer therapies and pediatric care are under influence of hospice situation • Ethical and legal regulations of pediatric hospice are not fixed or well-defined	• Guidelines and instructions should be developed exclusively for hospice in pediatric oncology • Both medical and legal authorities in each jurisdiction should be involved in designing these guidelines • Autonomy of the patient without age-related bias should be considered	^110,111,113,117,119^
Quality improvement	• Several parameters in quality assessment of hospice are important including utilization of chemotherapy and invasive procedures, admissions, distressing symptoms and cost • Several methods have proposed for quality measurement in pediatric oncology hospice • Standards of optimization have not been elucidated in this field	• Key elements and their level of quality should be defined at the beginning of establishment of hospice in this setting • Decreasing the pain, improving family closure and harnessing the private and public expenses should by considered in this field • Standard methodologies should be applied to evaluate the success of hospice services in each center, periodically	^39,71,128,130,132^

### General Considerations for Pediatric Hospice Care

There is adequate level of evidence in medical literature that proves the necessity of HC in oncology. It has been shown frequently that hospice philosophies are beneficial for the patients, caregivers and health care system. A majority of this evidence is derived from adult-based studies that compared outcomes of patient referrals to hospice services to the outcomes of staying in general or intensive care units. ^27-29^ As mentioned before, the boundaries and definitions of hospice/palliative care is not well defined. Hence, establishing clear-cut points in the continuum of concurrent care transitioning from palliation and curative measures into hospice and EOL care is essential for improving the quality of life and death in this specific population. However, physiologic, emotional, and social characteristics of children bear specific requirements that mandates exclusive services.^30^ Due to lack of exclusive experience in pediatric hospice, many of the currently implemented approaches are derived or copied from adult guidelines.

In a study on a diverse range of advanced cancer patients, it was shown that lower age was associated with longer survival time in hospice setting. ^31^ Such facts dictate a specialized planning for PHC. The first and most important role of hospice is changing “pain and distress” with “joy and closeness” for the patient and the family.^32^ Therefore, finding the earliest but most conservative time to start the service can be immensely beneficial for the patients, families, and healthcare system.^33-35^ This can be accomplished through defining the indications or eligibility criteria,^36^ setting the priorities, addressing symptoms such as pain, nausea, vomiting, loss of appetite, respiratory failure, tiredness, anxiety, or depression,^37^ and decision-making about location and level of care,^38^ quality standards,^39^ modified expectations,^40^ and logical and legal issues.^41^

### Demography

Knowing the statistics and demography of children who used PHC versus non-users can be helpful in long-term policy making for improving the establishment of this service as a quality-of-life-saving strategy. It is notable that some minorities might have lower knowledge or accessibility to these services.^42^ In a study in USA from 2011 to 2013, factors like non-Hispanic White race, complex chronic comorbidities, mental issues, technology dependence, specific tumors, and residence in rural areas with lower socioeconomic environments were considered as hindrance factors to hospice admissions.^43^ In another study in 2013, it was shown that race, ethnicity, payor status, patient diagnosis, and religion are significantly associated with hospice enrollment and its outcome.^44^ Cancer type, hospital size, insurance status and geographic location are other defined factors in this field.^45^

Considering the value of home-based care in pediatric hospice, some predictive demographics can be found among the above-mentioned factors to forecast the risk for hospital death. While data in this regard for pediatric oncology hospice is very limited, information from other child-oriented hospice settings can be useful. Age lower than a year, presence of congenital causes, certain locations, proximity to tertiary hospitals are associated with dying in hospital.^46^ Focusing on pediatric intensive care unit (PICU) death rates among children with cancer, risk factors such as Hispanic ethnicity, hematologic malignancies, hematopoietic stem cell transplantation, cumulative number of PICU admissions, receiving cancer therapies during the final month, and delayed palliative care involvement have been discovered.^34^

In our data pool for this review, we detected a variety of demographic features for pediatric cancer patients under hospice care. The information derived from the references included data for age groups (neonates, children, and young adults),^31,33,47-49^ disease type and conditions,^28,29,31,34,35,50-52^ types and levels of medical interventions,^33,35,51,53-56^ access to specialized care and sufficient communication,^57-61^ geographical location,^43,45,49,62-64^ physical, mental and behavioural comorbidities,^43,63,65,66^ gender,^25,31,43,67^ previous hospitalization unit or ward,^45,49,68,69^ health insurance type,^42,45,60,70^ clinical phase (stable, unstable, deteriorating, terminal),^71,72^ cost of care,^60,73^ race and ethnicity,^42,74,75^ financial background,^43,75^ familial characteristics and siblings,^76^ cultural background,^44,77^ number of admissions,^53^ access to palliative care before hospice,^35,78^ symptomatology,^37,52,79^ and access to general hospitals.^45^ It should be noted that some of the studies included non-pediatric-cancer populations, as well. Some of these parameters were associated with different outcomes and success rates of hospice services, which are discussed in different sections of this review. Based on the findings it can be assumed that middle categories of age, non-hematologic malignancies at lower stages, receiving fewer intensive therapies, absence of comorbidities, access to insurance coverage, advanced care and education, less frequent history of admissions and utilization of palliative care are associated with better outcomes regarding the EOL quality of life (QOL) and hospice attendance or success.

### Parents and Family Members

Parents are major determinants for initiation, process, place, and quality of hospice care in pediatric settings.^57^ In such complicated situations, parents are the first-line legal authorities to adopt this kind of lifestyle for their loved ones.^80^ This produces is a huge burden of mental stress for them that can threaten their efficient critical-thinking and proper decision making. It has been shown that these parents, suffer more from psychiatric disorders, including anxiety and depression compared to general population.^81^ These guardians have to plan their caregiving strategy, deal with financial issues, and handle the familial and social complications of entering a hospice-associated life.^82^ This fact is even more complicated among the families that have children with cancer, both due to debilitating expenses and bombardment with every-day advertised experimental and uncertain therapeutic options.^83^ One of the greatest challenges for parents is digesting the dilemma, when their decision is not in accordance with their dying child; this makes it even more difficult for the healthcare team to reach a certain opinion, as well.^84^ These unwanted and unplanned consequences, plus feelings of regret, continue even after their great loss, and can be accompanied by prolonged grief and disturbed QOL.^81^ The role of hospice in family care is to ease the pressure and remove this burden.

Taking care of parents of children with terminal illnesses is as important as paying attention to the patients. After the diagnosis, there is a huge risk for total collapse of personal, financial, and mental health of a family dealing with the impending death of a younger family member.^77^ The most common aspects of supportive needs among parents have financial, psychosocial, and legal sources.

Besides the patients and their parents, other family members deserve to be acknowledged in this process. Young brothers and sisters are priorities in this regard due to their vulnerable situation and minor authoritative role in this family hazard. A literature review by Ridley and Frache demonstrated that there is an alarming gap in coverage of care for youngster siblings of deceased children.^85^ Several approaches have been developed in cancer hospice settings, due to their higher prevalence. Group sessions, recreational camps and educational activities have been tried for bereaving children with promising results, but availability of such facilities has not been globally established.^85^ In addition, the role of young siblings as assisting caregivers in hospice has been neglected, almost completely.^86^

It can be concluded that concordant sponsorships from medical, social, and governmental entities are required to comprehensively support families and decrease the ravaging effects of this issue on parents and other family member.

### Psychosocial Issues

Psychological and social aspects of PHC are as important as its somatic features.^87^ The process of providing PHC starts with this portion of care. In recent years there has been a major change from the state of disappointing expectations from cure-oriented thinking that may have a destructive effect on the EOL management procedure, to optimism in a variety of available options for improving the near-death trajectory of a child and the family.^76^ This mindset will boost their mental and spiritual strength, significantly.

After building a clear and honest bond, hospice systems should provide the most peaceful environment to control and ease the understanding of the end and grief for parents, siblings and caregivers.^76^ Even the decisions about the array of medical interventions in this phase should be finalized based on the emotional and relational responses from the care recipients.^88^ This issue is very critical in cancer settings in which lots of curative and palliative interventions are available. Then they should provide bereavement aid including emotional support, recognition of abnormal and pathologic responses, and follow-ups.^76^ These maneuvers generally target parents or caregivers, rather than their young patients. However, social interactions of a dying child should be addressed as a very complicated issue, as well. In order to plan effectively, determinants of psychological risks should be defined. Studies have shown that besides a rewarding and meaningfulness feeling, caregiving is commonly accompanied by adversities like anxiety, depression, intensified grief reactions, physical and mental health dysfunction and even, increased caregiver mortality ^89^ compared to general population of parents or those with children affected by non-terminal illnesses. With the use of indicators such as relationship with the patient, age at caregiving, accessibility of help, financial support, and history of loss, high risk entities can be more feasibly detected and supported through psychosocial back up programs in pediatric cancer hospice.

Reports from UK childhood cancer centers shows that there are multiple areas for providing these kinds of supportive measures but generally no firm standard has been established to make them continuously and comprehensively accessible. These areas include education, staffing, facilities for children and families, and psychosocial services in terms of group activities and transition plans.^90^ In this way, presence of psychiatrists and psychologists can be undeniably beneficial regarding they role in implementation of specialized assessments, diagnosis, interventions and research, both for the child and the family.^91,92^ However, due to lack of extensive experience in this field the true value of these collaborations has not been fully elucidated in PHC.

In addition, death education, initiated in schools can have a preventive effect on mental health disorders for families who will expose to such tragedies, unexpectedly.^93^ Continuing this type of education through the society with meticulously supervised methods can act as a public immunization and enhance the society with self-care tools to decrease the devastating consequences, from before entrance to the trajectory of a child’s death.^94^

At the end it should be stated that this harsh pathway is more impassable to go through alone. Therefore, the value of mentoring and support groups from those who had the same experience cannot be overemphasized.^95^ In this pathway, instead of being considered as hopeless, the patient and the family will be hopeful for spending nice and memorable days together. Families will enjoy the memories of those valuable last days to harness their bitter bereavement. Also, the role of faith which has been introduced in adult settings, should be further explored in PHC, as well.^66^

### Budgeting and Insurance

EOL care is associated with a huge financial burden, both for the involved family and the supporting heath care system. These budgeting issues are powerful to the extent that they can change the pathway of care for a dying child. Decreasing the amount of expenses for pointless therapies or unnecessary admissions is hidden in the philosophy of PHC to facilitate the passage of the family through this harsh course. It has been shown in adult settings that implementation of HC in the form of home based care is significantly more cost-saving than hospital-located conventional care.^60^ However, the hospice process can be very expensive by itself. Therefore, financial pre-planning for critical situations is mandatory. Logically, families cannot actively be involved in the investment procedures for these plans, especially if the time period between the diagnosis and EOL situation is short. EOL insurance is not a common or routine investment among families who cannot predict such unexpected grave occasions.^96^ Also, decision making in this situation about the EOL plan can be immensely difficult.^82^ Hence, governmental authorities, social organizations, insurance companies and finally health care systems have to be prepared for providing this kind of support. Regarding the fact that PHC is still a newborn science and entangled with the multi-aspect complications of pediatricEOL services, the rubrics of budgeting and financial planning for it has not been fully developed in many countries. However, there are a limited numbers of studies that tried to explore this new field and propose guidelines. The first step for establishment of an effective financial plan is recognition of the sources of cost. This can be achieved through cost-evaluation studies. Such studies have proposed that the intensity of treatment protocols, physician care, nursing care and presence of some clinical symptoms including asthenia, anorexia, bedsore, nausea and vomiting, can result in rising costs.^73^

There is a considerable risk that governments do not have the capacity to provide for all the hospice costs. In this way, the role of support from charities will be vital. Nowadays, a considerable amount of national hospice expenses in countries like England and Wales is provided by charities for PHC.^97^ However, this might persuade the government to step back from their true supportive stands. Additionally, it should be noted that governments may devote a considerable budget to HC but recognition of a suitable share for PHC needs more organized amendments.

Insurance organizations play a great role in handling of PHC costs. Insurance coverage can be considered as a determinant in choosing the strategic style for the EOL care.^70^ Generally, public governmental insurance plans cover these types of expenses. Therefore, those patients who are covered only by the private ones might be deprived of such services. There are territory-based local eligibility criteria for utilization of hospice insurances. For example a certificate from an accredited practitioner to announce that the patient is within the terminal 6 months is mandatory, in some settings.^47^ All these diversities in coverages can affect the delivery of PHC. Different jurisdictions have different insurance coverage policies for hospice from zero to 100% of the expenses.^16,98^ Also, there is a difference between coverage for hospice and palliative care in some places and the policies are evolving. For example, in an American setting, palliative care is paid by personal insurances, involving limitations such as standard co-pay and deductions. A partial coverage is also provided by Medicaid and Medicare. But for hospice, expenses are fully paid by Medicaid or Medicare and coverage includes medications, medical equipment, nursing care, social and spiritual services. The same is true with certain personal insurances.^23^

### The Place for Death

One of the key features of PHC is the place in which the young patient passes away. This issue has been the focus of a considerable portion of studies. In fact, the matter of location for spending the last days can be the leading determinant for planning the strategy of HC.

HPC services can be delivered at different locational or strategic points. Supervised home care is one of the most appealing approaches.^99^ Specialized hospice wards are also available in limited numbers that provide a combination of medical and spiritual care.^100^ In resource limited situations, as a last resort, regular, pre-critical, or critical care units can be adjusted, as much as possible, to accept the young patients for this purpose.^38^ In addition, medical procedures are dispensed at different levels of intensity.

Serving the patient and family with hospice alone is one option. Due to scarcity and costly nature of these services in some places, simple private nursing care is substituted on some occasions, generally with low satisfaction rates. Another option for the pediatric patients is admission in “concurrent” HC.^101^ Although, concurrent palliation is a better terminology in this regard, due to continuation of therapeutic and curative measures.^101^ In this setting the patient can be benefitted from both life prolonging care and pain reducing hospice.^63^ Because this approach is more resource consuming, developing certain criteria for its recipients is fundamental.

The decision about where a patient passes away is affected by numerous factors from different entities of this challenge. Each one of the patient, family and medical team may have a divergent opinion about it. The usual available options include home, general wards, critical care or emergency settings, general hospice, and pediatric hospice facilities. For children with cancer, oncology wards are also a common place to die. However, accessibility to these choices varies in different geographical zones.^64^ In addition, continuation of hospitalization in the regular oncology or pediatric wards cannot be recommended.

It has been shown that home is the most appealing place for the ending days, both from parents and clinicians’ point of view.^62^ Statistical data shows that this happens for a majority of patients under PHC, as well.^68^ Although, the actual and the preferred location can be incongruent, specifically for children with haematologic malignancies.^62^ On the other hand, presence of an active palliative hospice care can increase the congruency. Notably, in some cases, parents showed more tendency to choose hospital-base care compared to specialized PHC institutions, compared to clinicians.^62^ This implies the lack of adequate introduction and popularization of PHC to the public audience. Establishment of HC teams in healthcare centers has pushed the curve toward home-based or hospice-based admissions.^102^

Studies demonstrated that cultural, racial, and clinical backgrounds of patients can alter the aforementioned assumptions. For instance, in study in UK that categorized the destinations into hospital and hospice or home, it was shown ethnic minorities were less likely to die in hospice, while a lower portion of White children died in the hospital setting.^75^

It should be noted that the optimal choice for the final place can be totally different case-by-case. But based on the aforementioned studies that considering home-death as a preferrable outcome, some arrangements highly improve the QOL and feasibility of this type of care for the involved families. The term “shared care” emphasizes a collaboration of family and the medical team during this period. ^75^ A wise approach would be educating the household members to handle the major requirements of caring to stay independent from frequent professional visits. The share of healthcare team in this situation is more educational, rather than therapeutic. This method both save the financial status of the bereaving family and spare the limited number of trained staff to attend more cases. In addition, the number of unnecessary admissions and hospital deaths can be diminished through such a goal-directed programming.^52^ Insurance policies and governmental financial strategies can also significantly change the trends in this regard.^103^ It should be kept in mind that providing hospice services is accompanied by environmental, cultural, economic and religious issues that need preplanning.^104^ Also, it is notable that some authors have argued against considering hospice as a location of care and they defined it as a philosophy of compassionate, multidisciplinary care that can be delivered at any place.^105^

### Specialists

As mentioned frequently in the previous parts, PHC is a teamwork, and it will be handled optimally if the whole team be present with adequate capabilities, expertise and numbers. Besides their pragmatic function, the accessibility to this team will be highly comforting for both the child and the stressed caregivers involved. It should be kept in mind that there is no specific hierarchical order for roles and duties in this procedure and the importance for all of the elements are totally overlapping. Considering the majority of studies in this field, nurses are the mainstays of this procedure for two reasons. First, their professional dexterity in handling every-day needs of hospice children makes them the frontiers of PHC team. Secondly, they are health practitioners that provide care at a high professional level with considerably longer continuation, due to the nature of their occupational characteristics and history of trainings.^106^ In this way, their persistent active presence will help a lot in management of a near death child and the family. On the other hand, lack of adequate nursing staff or availability of home visits significantly decrease the quality of lives of the challenged household in that terminal hectic stage.^107^ Physicians are also critical in easing the last days with helping to relieve the pain, giving medical consultations and guiding the caregivers for an optimized decision making. Palliative and hospice care specialists are valuable agents in PHC, but regarding their scarcity, most centers lack the access to such ideally trained professionals. Considering the fact that majority of children on hospice settings are transferred from specialized care services, usually the in-charge physicians are doctors with specialties and subspecialities. Oncology fields are the major referring wards for pediatric hospice; therefore, oncologists have the highest exposure and experience with these cases. Stem cell transplant specialists also are in contact with a big population of children with rare and terminal diseases.^48^ However, pediatricians and oncology pediatricians are the most eligible clinical entities in this regard, with deep insight into somatic, psychological, and social features of PHC.^59^ Other practitioners like surgeons and anaesthesiologists also have positive effects.^108^ In addition, the presence of mental health clinicians such as psychologists and psychiatrists are non-negotiable in preparing the child and family for their destiny. An optimal example in this regard is an organized team involving pediatric oncologists, palliative care physicians, HPC trained nurses, psychologists, child life specialists, and medical social workers.^58^ Also, pharmacists can improve the quality of medication management in pediatric EOL care.^109^ The last but not least group involves social workers, spiritualists, lawyers, and other non-clinical support coordinators to help the parents navigate socio-economic-legal challenges. This list can be elongated with more clinicians and non-clinical supporters but considering the sensitivity of this issue, empathy and moral devotion are the main requirements of being a team member in hospice services for children and their caregivers.

### Regulations

Considering the fact that the whole procedure is dealing with life of a person who can be the most valuable belonging of an entire family, every single act or even comment will bear a huge burden of legislative responsibilities for the PHC team.^110^ Ethical regulations for EOL care in pediatrics is considerably debatable, fluid and sometimes ambiguous. This fact stems from the uncertainties about the principle of autonomy in this age group. Physical, mental and social development and the chance for survival into adolescence can be different for children with the same age.^111,112^ Therefore, in some territories there is no designated age limit for eligibility to consent to, or refuse a medical approach.^113^ In addition, cultural background affects the criteria of capability and role in consenting for child and the caregiver in such procedures.^114^ In many cases there is no specific eligibility criteria for pediatric hospice and the rules for adults are being implemented. Because HC is not a curative therapeutic approach, instead of “indications”, guidelines usually use the term “eligibility” criteria. However, these criteria are usually conditional and negotiable in hospice field, rather than fixed and strict rules.^36^ This matter is even more ambiguous in PHC. In place of indication or eligibility, the American academy of pediatrics have proposed “trigger points” for initiation of such EOL services. These include severe fetal or traumatic disabilities, a progressive or unresponsive disease state with poor prognosis or therapy-resistance, prolonged or frequent hospitalizations, and reaching to certain ages for decision making strategies.^115^ For pediatric cancer patients, globally or even nationally applicable standards are very limited and local criteria is usually established by private institutions. Some centers advocate for accessibility to EOL care regardless of the diagnosis,^116^ while disease-based prioritization might be necessary in resource limited settings. Considering the broad spectrum of needs of pediatric hospice patients, selection and prioritization of major symptoms for treatment would be necessary. Addressing pain is one of the top-rank common priorities.^61,79^ Amelioration of nausea, vomiting, loss of appetite, respiratory failure, tiredness, anxiety, and depression should also be adequately managed. In a study in pediatric cancer patients, bleeding, dyspnea, pain, seizures, and delirium were reported to be the most prevalent symptoms during the last days of life, so, addressing these discomforts may be prioritized in oncology hospice settings.^37^

Eligibility criteria for receiving this sub-specialized and multi-compartmental intervention should be defined. Each and every hospice center has its own local admission parameters and scoring systems. The format and availability of these criteria is divergent based on cancer type, as well. Exclusive guidelines for pediatric patients in cancer setting has not been clearly provided.^117^ Some general instructions which are developed by insurance authorities include being terminally ill with a medical prognosis of 6 months or less and consenting for hospice limitations. One of the key issues and trigger points in hospice care is the “do not resuscitate” or “DNR” code.^118^ This code had been a prerequisite in some centers for hospice, but nowadays EOL care receivers can choose their demand for resuscitation. While having an official DNR code is not a universal amenity, in countries with developed health care systems including Canada and USA, signing a DNR agreement is no longer mandatory for hospice services.

There are clinical practice guidelines to guide the initiation and progression of hospice/palliative care in oncology.^119^ Unfortunately, the recommendation have not been tailored for pediatric settings but they generally include endorsement for early initiation of palliative care process (not hospice), referrals to specialized teams, use of scoring systems for performance status evaluation, and hospice consideration for those with less than 6 months prognosis.^119,120^ For palliative care (not hospice alone), usually the guidelines are more detailed and provide criteria for both on-diagnosis and during-the-treatment in pediatric oncology setting.^121^

As a common point in this area, pro-active legal involvement of the patient, surrogate decision-makers or parents and the healthcare providers is recommended.^72,122^ Neurobiological (instead of chronological) parameters of adulthood have been suggested as a practical way of assessing a child’s capability for consenting in such situations^123^ but this requirs more evaluations. One of the most neglected groups in HC fields includes adolescents and young adults (AYAs). Considering their maturity state, this population needs to be more involved in decision makings.^124^ Due to their borderline aging category, a lack of recognition and subsequent heterogeneity for their management is very common.

Furthermore, after death of a child the process may continue with more intense issues like requirement for an autopsy.^125^ Hence, involvement of a lawyer or forensic physicians has been endorsed in studies.^126,127^

### Quality Improvement

Pediatric hospice is a recent concept which needs continuous trimming and reassessment. Methodologies of investigation in pediatric hospice/palliative care are growing and new approaches are under exploration to address the specific needs in this setting.^128^ The efficiency and satisfactoriness of these services can be scientifically measured through some standardized tools.^39^ Patient and family reports, staff observations and retrospective data analysis are highly valuable resources for these evaluations.^129^ Unfortunately, exclusive data to define optimum quality of care in pediatrics is not as accessible as adults. Some evaluation determinants which have been derived from interviews from PHC oncology settings include communication and guidance, inclusion of interdisciplinary professionals, symptom-based measures, and considerations for family preferences.^130^

Interestingly, late-stage chemo-radiation with the goal of symptom relief can be considered as a part of palliation.^78^ However, the decision in this direction should be debated with highest attention in order to prevent negating the value of painless EOL care.^131^ In fact, continuation of anti-cancer therapy in children has been announced as one of the key causes of delay or deprivation of children in need of PHC.^51^ In addition, maximization of the parent-children closure time has been announced as the number one priority in some observations.^132^ Notably, goal-directed programs for education of clinical staff including physicians and nurses for hospice has shown improvement in PHC success rates.^54,55^ Also, availability of hospice services in the same clinical institution of admission for the primary disease can boost efficiency.^69^ Therefore, a risk-benefit analysis is required before strategic planning in this regard.

In a quality measurement study in 2022 on pediatric cancer cases from multiple centers in the United States parameters such as utilization of chemotherapy, mechanical ventilation, intensive care units, and counts of reported distressing symptoms in the last 30 days of life were checked. It was shown that a great proportion of non-hospice users were among the families with lower income. This means that they chose more costly and distressing care approaches for their children.^53^ Based on these reports, we can state that there is a huge gap for optimization of PHC. Hence, quality improvement with a socio-educational perspective should be considered as a constant attachment of hospice in achieving the desired goals, in a more time- and cost-efficient manner. Development of high-tech methods such as artificial intelligence has opened new horizons for more strict decision making and referrals to hospice care. However, deep emotional aspects of this issue prohibits carefree establishment of machine-guided approaches in PHC.^133^

As the last step, periodical evaluations are required for assessment of trends in this evolutionary discipline. It is obvious that data obtained from care recipients including patients and families can be very enlightening for success evaluation of PHC teams and facilities.^71^ These evaluations can be implemented both for general strategic areas of PHC or for detailed issues such as personnels’ eligibility, knowledge and skills.^134^ Regarding the short history of modern PHC implementation in the whole world, involvement and assistance of experienced specialists from adult hospice services can be constructive for upgrading the PHC procedures.^135^

## Conclusion

Our findings highlight that our understanding and delivery of pediatric hospice resembles an ocean of uncertainties, doubts and unknown issues. Regarding the maturation of knowledge, this field of medicine and patient care is in its infancy. This immaturity makes reaching a consensus on guidelines with straightforward instructions seemingly impossible. While the majority of the current rubrics in this field are generalizable to a diverse spectrum of ailments, individualization for each group is clearly required. Among these groups pediatric cancer patients are exclusively considerable due their huge population and special needs. However, as mentioned before, the complications of hospice care in pediatric oncology include lack of child-oriented guidelines, psychosocial issues, challenges of parents and other family members, uncertainties for the place and intensity of care including continuation or discontinuation or modification of cancer therapies, financial obstacles, inadequacy in the number of specialized practitioners and ambiguity of monitoring parameters for quality improvement. The same can be said for all other life-limiting conditions experienced by children and their families. Each and every one of these issues requires a well-targeted approach for comprehension, analysis and research to find evidence-based and logical solutions for easing the physical, mental, emotional and spiritual pain of the involved entities in a child’s final palliative and end of life journey.
